# Role of source control in critically ill candidemic patients: a multicenter retrospective study

**DOI:** 10.1007/s15010-024-02222-z

**Published:** 2024-03-12

**Authors:** Markos Marangos, Petros Ioannou, Laurence Senn, Anastasia Spiliopoulou, Sotiris Tzalis, Fevronia Kolonitsiou, Maria Valta, Sofia Kokkini, Jean-Luc Pagani, Dimitra Stafylaki, Fotini Paliogianni, Fotini Fligou, Diamantis P. Kofteridis, Frédéric Lamoth, Matthaios Papadimitriou-Olivgeris

**Affiliations:** 1 Division of Infectious Diseases, University General Hospital of Patras, Patras, Greece; 2 Department of Internal Medicine, University General Hospital of Heraklion, Heraklion, Greece; 3 Infectious Diseases Service, Lausanne University Hospital and University of Lausanne, 1011 Lausanne, Switzerland; 4 Department of Microbiology, University General Hospital of Patras, Patras, Greece; 5 Division of Anaesthesiology and Intensive Care Medicine, University General Hospital of Patras, Patras, Greece; 6 Department of Intensive Care Medicine, University General Hospital of Heraklion, Heraklion, Greece; 7 Department of Adult Intensive Care Medicine, Lausanne University Hospital and University of Lausanne, Lausanne, Switzerland; 8 Department of Clinical Microbiology and Microbial Pathogenesis, University Hospital of Heraklion, Heraklion, Crete Greece; 9 Institute of Microbiology, Lausanne University Hospital and University of Lausanne, Lausanne, Switzerland; 10 Infectious Diseases Service, Cantonal Hospital of Sion and Institut Central des Hôpitaux (ICH), Sion, Switzerland

**Keywords:** *Candida albicans*, Catheter removal, Source control, Antifungal treatment, Intensive Care Unit, Sepsis, SARS-CoV-2

## Abstract

**Purpose:**

Candidemia is associated with high mortality especially in critically ill patients. Our aim was to identify predictors of mortality among critically ill patients with candidemia with a focus on early interventions that can improve prognosis.

**Methods:**

Multicenter retrospective study.

**Setting:**

This retrospective study was conducted in Intensive Care Units from three European university hospitals from 2015 to 2021. Adult patients with at least one positive blood culture for *Candida* spp. were included. Patients who did not require source control were excluded. Primary outcome was 14-day mortality.

**Results:**

A total of 409 episodes of candidemia were included. Most candidemias were catheter related (173; 41%), followed by unknown origin (170; 40%). Septic shock developed in 43% episodes. Overall, 14-day mortality rate was 29%. In Cox proportional hazards regression model, septic shock (*P* 0.001; HR 2.20, CI 1.38–3.50), SOFA score ≥ 10 points (*P* 0.008; HR 1.83, CI 1.18–2.86), and prior SARS-CoV-2 infection (*P* 0.003; HR 1.87, CI 1.23–2.85) were associated with 14-day mortality, while combined early appropriate antifungal treatment and source control (*P* < 0.001; HR 0.15, CI 0.08–0.28), and early source control without appropriate antifungal treatment (*P* < 0.001; HR 0.23, CI 0.12–0.47) were associated with better survival compared to those without neither early appropriate antifungal treatment nor source control.

**Conclusion:**

Early source control was associated with better outcome among candidemic critically ill patients.

**Supplementary Information:**

The online version contains supplementary material available at 10.1007/s15010-024-02222-z.

## Introduction

Infections caused by *Candida* spp. are frequently encountered among patients admitted to Intensive Care Units (ICU). According to the second Extended Prevalence of Infection in Intensive Care (EPIC II) study, *Candida* spp accounted for 19% of all infections in Europe [1]. Over recent decades, the epidemiology of candidemia has undergone a significant shift characterized by an increase in its incidence and a rise in the prevalence of *C.* non-*albicans* species [2, 3]. In Europe, *C. albicans* still remains the most commonly isolated species in central or northern countries, while *C. parapsilosis* has become predominant in southern regions [2, 3]. The incidence of candidemia also saw a rise in some countries during the Coronavirus Disease 2019 (COVID-19) pandemic [4–6].

Candidemia poses a significant threat to critically ill patients and is associated with high mortality rates, especially among those with septic shock [3, 7, 8]. Adequate and timely antifungal treatment plays a pivotal role in patient survival, with echinocandins being recommended as the preferred choice according to current guidelines [8–14]. In addition, prompt source control, like catheter removal in catheter-related candidemia or drainage of abscesses in intra-abdominal candidiasis, has shown varying results in reducing mortality in previous studies [11–19]. However, the combined effect of these interventions (antifungal treatment, source control) on mortality has not been extensively explored [7, 11–13, 17, 20, 21].

In a prior study conducted at Lausanne University Hospital, the impact of source control, particularly catheter removal, on candidemia outcomes in patients with sepsis or septic shock, was demonstrated [20]. To further investigate the significance of timely source control and identify other potential predictors of mortality, our multicenter study aimed to validate these findings, focusing on critically ill patients with candidemia across two European countries, Greece and Switzerland.

## Materials and methods

### Study design

This retrospective multicenter study was conducted during a 7-year period (2015–2021) at three tertiary hospitals: the University General Hospital of Patras (UGHP) and the University General Hospital of Heraklion (UGHH) in Greece, and the Lausanne University Hospital (LUH) in Switzerland.

### Patients

Inclusion criteria included: adult patients (≥ 18 years old), at least one positive blood culture set for a *Candida* spp., and admission in ICU within 48 h from candidemia onset. Patients who did not require source control were excluded. The primary outcome was 14-day mortality, and the secondary one was 30-day mortality. Data on demographics, comorbidities, septic shock, antifungal treatment, source control procedures, decisions regarding care withdrawal, and outcomes were collected. Infectious diseases specialists conducted daily rounds in all three ICUs during the study period.

*Candida* species were identified by Vitek-2 YST card (bioMerieux, Marcy l’Etoile, France) in UGHP and UGHH and by matrix-assisted laser desorption ionization time of flight (MALDI-TOF) mass spectrometry (Bruker, Billerica, MA) in LUH. Antifungal susceptibility testing in UGHP and UGHH was performed by Etest (bioMérieux) on RPMI-2% glucose agar, and by microbroth dilution method (Sensititre YeastOneTM, Trek Diagnostics Systems, ThermoFisher Scientific, Cleveland, OH) in LUH. Results of minimal inhibitory concentrations (MIC) were interpreted according to the Clinical and Laboratory Standards Institute (CLSI) clinical breakpoints [22]. Beta-d-glucan was available in the LUH since 2017.

### Definitions

Candidemia onset was defined as the date the first positive blood culture was drawn. We regarded a new episode to have occurred when more than 30 days had passed since the first negative blood culture from the initial episode. Septic shock followed the Sepsis-3 International Consensus definition [23]. Catheter-related candidemia was defined per IDSA guidelines, either by a positive catheter tip culture showing the same organism as in the candidemia (across all hospitals), or by a positive differential time to positivity favoring the blood culture drawn from the catheter (only in LUH) [24]. In all three hospitals, catheter insertion was guided by echography, and specific protocols were in place to address catheter-related infections. Appropriate antifungal treatment was defined as administrating an antifungal agent, for which the isolate was defined as susceptible according to CLSI criteria [22], at an adequate dosage and diffusion in the infection site. Source control was warranted for catheter-related candidemia (removal of all intravascular catheters), candidemia of unknown origin (removal of all intravascular catheters) intra-abdominal infection (surgical or imaging-guided drainage of abscess, peritoneal collection), obstructive urinary-tract infection (removal of obstruction), endocarditis (valvular replacement). We used the cutoff of 72 h to define early interventions (antifungal treatment initiation, source control) from candidemia onset, which corresponded to the usual time to positivity of *Candida* spp. in blood cultures [20]. Patients were considered to be on maximal care until a decision of treatment withdrawal or instauration of palliative care has been documented in the medical record.

### Statistical analyses

Data analysis utilized SPSS version 26.0 (SPSS, Chicago, IL, USA). Categorical variables were analyzed with Chi-square or Fisher exact test and continuous variables with Mann–Whitney *U* test for 14-day and 30-day mortality as the dependent variables. Covariates were tested for multi-collinearity through variance inflation factor assessment; those with *P* < 0.1 in the univariate analysis and not collinear were used in multivariate analysis. After checking Cox assumptions, two multivariate Cox proportional hazards regression models were performed with 14- and 30-day mortality as the time-to-event. Hazzard ratios (HRs) and 95% confidence intervals (CIs) were calculated to evaluate the strength of any association. All statistic tests were two-tailed and *P* < 0.05 was considered statistically significant.

Kaplan–Meier curves of the survival probability of patients with candidemia that survived for at least 72 h according to early appropriate source control and early appropriate antifungal treatment were performed, with patients being divided in four groups:

- Group 1: neither early source control nor early appropriate antifungal treatment
- Group 2: only early appropriate antifungal treatment
- Group 3: only early source control
- Group 4: early source control and early appropriate antifungal treatment

Kaplan–Meier curves of the survival probability were performed in the subgroups of patients with candidemia of unknown origin, catheter-related candidemia, presence of septic shock. Since it was previously suggested that source control could be influenced by care withdrawal [25], Kaplan–Meier curves were performed among patients that were alive and in maximal care for 7 days after candidemia onset to assess the role of early source control on survival.

## Results

**Fig. 1:**
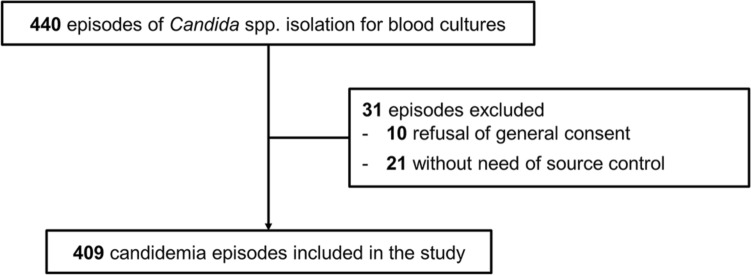
Flowchart of included patents

Of the 443 identified candidemia episodes, 409 episodes in 382 patients met the inclusion criteria (UGHP: 226, UGHH: 92, LUH: 91) (Fig. 1). A total of 414 *Candida* strains were isolated (2 different species were isolated in 5 episodes). *C. parapsilosis* was identified as the most prevalent species (181; 44%), followed by *C. albicans* (119; 29%), *C. glabrata* (57; 14%) and *C. tropicalis* (41; 10%) (Table 1). Sixteen isolates (4%) belonged to other *Candida* spp. *C. parapsilosis* was the most common species isolated in UGHP (60%) and UGHH (45%), while *C. albicans* predominated in LUH (60%). According to CLSI criteria, 181 (44%) isolates showed resistance or dose-dependent susceptibility to fluconazole (UGHP: 61%, UGHH: 22%, LUH: 26%), 35 (9%) were resistant or intermediate to at least one echinocandin (UGHP: 14%, UGHH: 1%, LUH: 3%), and 6 (2%) to amphotericin B (UGHP: 1%, UGHH: 0%, LUH: 3%). In the LUH, beta-D-glucan was performed in 28 (30%) episodes and was positive in 27 (96%). Eighteen episodes (4%) were acquired in other hospitals departments within 48 h from ICU admission.

**Table 1:** Patients’ characteristics

	LUH (*n* = 92)		UGHH (*n* = 91)		UGHP (*n* = 226)		All episodes (*n* = 409)	
Demographics								
Male sex	59	64%	61	67%	155	69%	275	67%
Age (years)	68	55–74	70	61–78	64	53–73	66	55–75
Age > 60 years	62	67%	69	76%	135	60%	266	65%
Co-morbidities								
Congestive heart failure	5	5%	13	14%	7	3%	25	6%
Chronic obstructive pulmonary disease	14	15%	21	23%	29	13%	64	16%
Diabetes mellitus	29	21%	21	23%	51	23%	91	22%
Chronic kidney disease (moderate or severe)^a^	12	13%	9	10%	14	6%	35	9%
Malignancy (solid organ or hematologic)	23	25%	18	20%	21	9%	62	15%
Obesity	25	27%	41	45%	45	20%	111	27%
Immunosuppression^b^	19	21%	6	7%	16	7%	41	10%
Charlson Comorbidity Index	4	3–6	4	3–5	3	1–5	3	2–5
Charlson Comorbidity Index ≥ 4	60	65%	54	59%	87	39%	201	49%
Microbiological data								
Prior episode of candidemia	3	3%	1	1%	23	10%	27	7%
Mixed bacterial/fungal bloodstream infection	11	12%	5	6%	13	6%	29	7%
Multiple *Candida* spp. isolated from blood cultures	1	1%	4	4%	0	0%	5	1%
*Candida* species (*n* = 426)								
*C. albicans*	55	60%	18	20%	46	20%	119	29%
*Candida* non-*albicans*	38	41%	75	82%	180	80%	293^c^	72%
*C. parapsilosis*	4	4%	41	45%	136	60%	181	44%
*C. glabrata*	17	19%	21	23%	19	8%	57	14%
*C. tropicalis*	8	9%	13	14%	20	9%	41	10%
Other *Candida* spp.^d^	9	10%	2	2%	5	2%	16	4%
Non susceptibility (resistance, intermediate or susceptible dose dependent)^e^								
Fluconazole	24	26%	20	22%	137	61%	181	44%
Echinocandin	3	3%	1	1%	31	14%	35	9%
Anidulafungin	1	1%	0	0%	13	6%	14	3%
Micafungin	3	3%	1	1%	28	12%	32	8%
Amphotericin B	3	3%	0	0%	3	1%	6	2%
Infection data								
Septic shock	52	57%	39	43%	82	36%	173	42%
SOFA score (points)	9	5–13	7	6–9	8	7–12	8	6–11
SOFA score ≥ 10 points	38	41%	17	19%	83	37%	138	34%
SARS-CoV-2 infection (prior month)	5	5%	16	18%	79	35%	100	24%
Prior antifungal treatment (prior month)	22	24%	74	81%	1402	62%	235	58%
Breakthrough infection^f^	13	14%	70	77%	131	58%	214	52%
Infection site								
Unknown origin	37	40%	53	58%	80	35%	170	42%
Catheter-related	25	27%	21	23%	127	56%	173	42%
Intra-abdominal	20	22%	2	2%	15	7%	37	9%
Urinary-tract infection	2	2%	11	12%	2	1%	15	4%
Other^g^	7	8%	4	4%	1	0.4%	12	3%
Management of candidemia								
Antifungal therapy initiated within 72 h	72	78%	78	86%	190	84%	340	83%
Echinocandin (*n* = 340)	50	69%	71	91%	136	72%	257	76%
Fluconazole (*n* = 340)	22	31%	5	6%	15	8%	42	12%
Liposomal amphotericin B (*n* = 340)	0	0%	2	3%	36	19%	38	11%
Appropriate antifungal therapy within 72 h	69	75%	75	82%	178	79%	322	79%
Type of targeted therapy								
Echinocandin	63	68%	67	74%	153	68%	283	70%
Fluconazole	12	13%	3	3%	15	7%	30	7%
Liposomal amphotericin B	2	2%	10	11%	44	20%	56	14%
Step down to fluconazole^h^ (*n* = 339)	23	35%	10	13%	0	0%	33	10%
Source control performed	74	80%	85	93%	211	93%	370	90%
Source control within 72 h	47	51%	73	80%	197	87%	317	78%
Care withdrawal within 7 days	13	14%	0	0%	0	0%	13	3%
Outcome								
14 days mortality	31	34%	27	30%	60	27%	118	29%
30 days mortality	36	39%	44	48%	94	42%	174	43%
ICU mortality	49	53%	61	67%	145	64%	255	62%

Data are depicted as number and percentage for proportions or median and Q1–Q3 for continuous variables

*LUH* Lausanne University Hospital, *SOFA* Sequential Organ Failure Assessment, *UGHP* University General Hospital of Patras, *UGHH* University General Hospital of Heraklion

^a^Defined as estimated glomerular filtration rate < 60 mL/min/1.73 m^2^

^b^Immunosuppression was defined as ongoing immunosuppressive treatment at infection onset, intravenous chemotherapy in the 30 days prior to infection onset, AIDS, neutropenia and asplenia

^c^two episodes had mixed candidemia by two different *Candida* non-*albicans* species

^d^*5 C. krusei, 4 C. lusitaniae, 3 C. kefyr, 2 C. dubliniensis, 2 C. guilliermondii*

^e^According to CLSI

^f^Breakthrough infection was defined as the occurrence of candidemia in a patient having received at least 3 consecutive days of systemic antifungal therapy

^g^5 empyema, 4 endocarditis, 3 deep surgical site infections

^h^For patients on targeted echinocandin or liposomal amphotericin B

Most candidemia episodes were catheter related (173; 42%), followed by unknown origin (170; 42%) and secondary to intra-abdominal infection (37; 9%). Septic shock was present in 173 episodes (42%). One-hundred (52%) episodes occurred in patients with SARS-CoV-2 infection (UGHP: 79; 63% UGHH: 16; 39%, LUH: 5; 21%).

Antifungal treatment was initiated early in 340 (83%) episodes (UGHP: 84%, UGHH: 86%, LUH: 78%) and it was appropriate in 322 (79%) episodes (UGHP: 79%, UGHH: 82%, LUH: 75%). Source control was performed in 370 episodes (90%), with 317 (78%) of them being performed early.

The 14-day, 30-day, and overall ICU mortality rates were 29%, 43%, and 62%, respectively. Antifungal treatment was administered in 390 (95%) episodes, with 340 (93%) receiving antifungal treatment within 72 h of candidemia onset; of these, 322 (95%) were considered appropriate. Care withdrawal within the first 7 days from candidemia onset was decided in 13 (3%) episodes. Table 2 displays the comparison of the characteristics of candidemia episodes between patients who survived and those who deceased within 14 days. In Cox proportional hazards regression model (Table 3), septic shock (*P* 0.001; HR 2.20, CI 1.38–3.50), SOFA score ≥ 10 points (*P* 0.008; HR 1.83, CI 1.18–2.86), and prior SARS-CoV-2 infection (*P* 0.003; HR 1.87, CI 1.23–2.85) were associated with 14-day mortality. On the other hand, the combination of early appropriate antifungal treatment and source control (*P* < 0.001; HR 0.15, CI 0.08–0.28), early source control without early appropriate antifungal treatment (*P* < 0.001; HR 0.23, CI 0.12–0.47) were associated with improved survival compared to those who received neither early appropriate antifungal treatment nor source control.

**Table 2:** Comparison of the characteristics of candidemia episodes between patients who survived and those who deceased within 14 days

	Survivors (*n* = 291)		Non-survivors (*n* = 118)		*P*
Hospital					
LUH	61	21%	31	26%	
UGHH	64	22%	27	23%	
UGHP	166	57%	60	51%	0.273^a^
Demographics					
Male sex	200	69%	75	64%	0.352
Age (years)	65	52–74	68	61–77	0.012
Age > 60 years	177	61%	89	75%	0.006
Co-morbidities					
Congestive heart failure	18	7%	7	6%	1.000
Chronic obstructive pulmonary disease	43	15%	21	18%	0.455
Diabetes mellitus	68	23%	23	20%	0.433
Chronic kidney disease (moderate or severe)^b^	23	8%	12	10%	0.442
Malignancy (solid organ or hematologic)	40	14%	22	19%	0.225
Obesity	81	28%	30	25%	0.713
Immunosuppression^c^	26	9%	15	13%	0.276
Charlson Comorbidity Index	3	2–5	4	2–6	0.023
Charlson Comorbidity Index ≥ 4	132	45%	69	59%	0.017
Microbiological data					
Prior episodes of candidemia	18	6%	9	8%	0.661
Mixed bacterial/fungal bloodstream infection	16	6%	13	11%	0.057
Multiple *Candida* spp. isolated from blood cultures	4	1%	1	0.8%	1.000
*Candida* species (*n* = 426)					
*C. albicans*	88	30%	31	26%	0.472^d^
*Candida* non-*albicans*	205	70%	88	75%	
*C. parapsilosis*	138	47%	43	36%	0.048^e^
*C. glabrata*	35	12%	22	19%	
*C. tropicalis*	25	9%	16	14%	
Other *Candida* spp.^f^	9	3%	7	6%	
Non susceptibility (resistance or intermediate)^g^					
Fluconazole	130	45%	51	43%	0.827
Echinocandin	24	8	10	9	0.839
Anidulafungin	10	3%	4	3%	1.000
Micafungin	22	8%	10	9%	0.839
Amphotericin B	3	1%	3	3%	0.361
Infection data					
Septic shock	93	32%	80	68%	< 0.001
SOFA score (points)	8	6–9	11	8–13	< 0.001
SOFA score ≥ 10 points	69	24%	69	59%	< 0.001
SARS-CoV-2 infection (prior month)	64	22%	36	31%	0.076
Breakthrough infection^h^	158	53%	61	51%	0.829
Infection site					
Unknown origin	119	41%	51	43%	0.740
Catheter-related	132	45%	41	35%	0.060
Intra-abdominal	18	6%	19	16%	0.004
Urinary-tract infection	14	5%	1	0.8%	0.078
Other^i^	7	2%	5	4%	0.339
Management of candidemia					
Antifungal therapy initiated within 72 h	252	87%	88	75%	0.005
Echinocandin (*n* = 340)	190	75%	67	76%	1.000^j^
Fluconazole (*n* = 340)	35	14%	7	8%	
Liposomal amphotericin B (*n* = 340)	24	10%	14	16%	
Appropriate antifungal therapy within 72 h	240	83%	82	70%	0.002
Source control within 72 h	263	90%	54	46%	< 0.001
Early appropriate antifungal therapy and source control					
None	4	1%	20	17%	< 0.001^ k^
Only early appropriate antifungal therapy	24	8%	44	37%	
Only early source control	47	16%	16	14%	
Both	211	74%	38	32%	

Data are depicted as number and percentage for proportions or mean and SD for continuous variables

*LUH* Lausanne University Hospital, *SOFA* Sequential Organ Failure Assessment, *UGHP* University General Hospital of Patras, *UGHH* University General Hospital of Heraklion

^a^Comparison of UGHP against both LUH and UGHH

^b^Defined as estimated glomerular filtration rate < 60 mL/min/1.73m^2^

^c^Immunosuppression was defined as ongoing immunosuppressive treatment at infection onset, intravenous chemotherapy in the 30 days prior to infection onset, AIDS, neutropenia and asplenia

^d^Comparison *C. albicans versus* non-*albicans*

^e^Comparison *C. parapsilosis versus* all other species

^f^*5 C. krusei*, *4 C. lusitaniae*, *3 C. kefyr*, *2 C. dubliniensis*, *2 C. guilliermondii*

^g^According to CLSI

^h^Breakthrough infection was defined as the occurrence of candidemia in a patient having received at least 3 consecutive days of systemic antifungal therapy

^i^5 empyema, 4 endocarditis, 3 deep surgical site infections

^j^Echinocandin *versus* both fluconazole and liposomal amphotericin B

^k^Neither early appropriate antifungal therapy nor source control as compared versus all other categories

**Table 3:** Cox proportional hazard multivariate regression of predictors of 14-day mortality of candidemia episodes

	*P*	HR (95% CI)
Age > 60 years	0.447	1.20 (0.75–1.94)
Charlson Comorbidity Index ≥ 4	0.676	1.09 (0.71–1.66)
Mixed bacterial/fungal bloodstream infection	0.249	1.43 (0.78–2.64)
*C. parapsilosis*	0.789	0.95 (0.64–1.41)
Septic shock	0.001	2.20 (1.38–3.50)
SOFA score ≥ 10 points	0.008	1.83 (1.18–2.86)
SARS-CoV-2 infection (prior month)	0.003	1.87 (1.23–2.85)
Intra-abdominal	0.967	0.99 (0.57–1.72)
Catheter-related	0.571	1.28 (0.75–1.70)
Early appropriate antifungal therapy and source control		
None	Reference	Reference
Only early appropriate antifungal therapy	0.732	0.91 (0.51–1.60)
Only early source control	< 0.001	0.23 (0.12–0.47)
Both	< 0.001	0.15 (0.08–0.28)

*HR* hazard ratio, *SOFA* Sequential Organ Failure Assessment

Supplementary Table 1 shows the comparison of the characteristics of candidemia episodes between patients who survived and those who deceased within 30 days. In Cox proportional hazards regression model (Supplementary Table 2), female sex (*P* 0.040; HR 1.39, CI 1.02–0.90), septic shock (*P* 0.031; HR 1.47, CI 1.04–2.07), SOFA score ≥ 10 points (*P* < 0.001; HR 2.00, CI 1.43–2.80), and prior SARS-CoV-2 infection (*P* 0.006; HR 1.65, CI 1.16–2.35) were associated with 30-day mortality. On the other hand, the combination of early appropriate antifungal treatment and source control (*P* < 0.001; HR 0.12, CI 0.07–0.21), early source control without early appropriate antifungal treatment (*P* < 0.001; HR 0.27, CI 0.18–0.39) were associated with improved survival at day 30 compared to those who received neither early appropriate antifungal treatment nor source control.

**Fig. 2:**
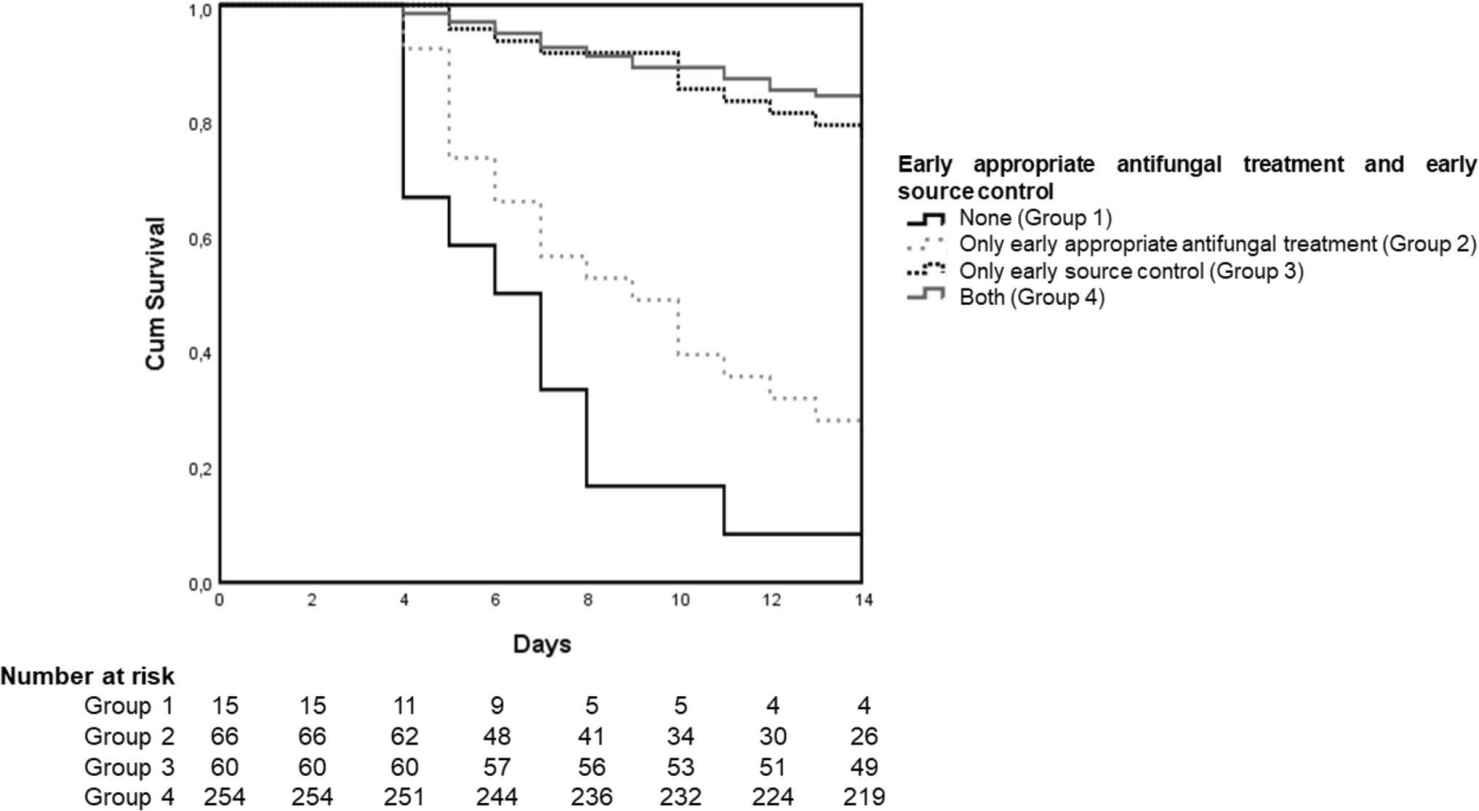
Kaplan–Meier curves illustrating the survival probability of episodes with candidemia based on early appropriate antifungal treatment and early source control in the 390 cases that survived at least 72 h from candidemia onset. Patients in Group 2 (those who received only early appropriate antifungal treatment) exhibited a similar outcome (*P* 0.120) to those in Group 1 (individuals who received neither early appropriate antifungal treatment nor early source control). However, both Groups 1 and 2 experienced worse outcomes (*P* < 0.001) compared to Groups 3 (patients with only early source control) and 4 (individuals who received both early appropriate antifungal treatment and early source control). There was no significant difference observed when comparing Groups 3 and 4

Figure 2 shows Kaplan–Meier curves illustrating the survival probability of episodes with candidemia based on early appropriate antifungal treatment and early source control in the 390 cases that survived at least 72 h from candidemia onset. Patients in Group 2 (those who received only early appropriate antifungal treatment) exhibited a similar outcome (*P* 0.120) to those in Group 1 (individuals who received neither early appropriate antifungal treatment nor early source control). However, both Groups 1 and 2 experienced worse outcomes (*P* < 0.001) compared to Groups 3 (patients with only early source control) and 4 (individuals who received both early appropriate antifungal treatment and early source control). There was no significant difference observed when comparing Groups 3 and 4.

Supplemental Fig. 1 presents Kaplan–Meier curves illustrating the survival probabilities of patients with candidemia in the following scenarios: (A) patients who remained under maximal care for 7 days following the onset of candidemia, (B) patients with candidemia of unknown origin, (C) patients with catheter-related candidemia, and (D) patients with septic shock.

## Discussion

The present study aimed to investigate predictors of mortality among candidemic patients requiring source control in three university hospitals, representing two regions with distinct epidemiological profiles, susceptibility patterns, and clinical management practices. Our findings underscore the paramount importance of source control, which emerged as the most influential factor affecting patient outcomes.

Consistent with previous publications, our study underscores the benefits of timely source control interventions, particularly catheter removal, in enhancing patient survival [11–15, 20]. This aligns with guidelines recommending early source control, although the debate surrounding its efficacy continues, driven by varying study outcomes and constraints in conducting randomized controlled trials [7, 9, 11–15, 18, 25–29]. One notable challenge is that catheter removal is not always feasible or safe, especially in cases of severe thrombocytopenia, administration of vasoactive drugs, or continuous renal replacement therapy. For instance, in a randomized trial on candidemia, only 51% of patients underwent early catheter removal, despite it being protocol recommended [30]. Therefore, retrospective observational studies such as ours, influenced by patient-specific factors, maximal care versus palliative approaches, and infection severity, face limitations in drawing definitive conclusions [25, 31, 32]. When considering patients on maximal care versus those with care withdrawal, it becomes evident that the absence of source control due to care withdrawal plays a significant role [7, 19, 25, 31]. To address this, we performed Kaplan–Meier curves among patients who remained alive and under maximal care for 7 days after candidemia onset, reaffirming the significant impact of source control on survival. It is worth noting that the participating hospitals exhibited heterogeneous management strategies, with Greek hospitals (UGHP: 87% and UGHH: 80%) more frequently performing early source control compared to LUH (51%). This disparity may be attributed to higher rates of catheter-related bloodstream infections in Greek ICUs [33]. In addition, the prevalence of *C. parapsilosis*, commonly associated with catheter-related candidemia, was higher in Greek ICUs than in LUH. Although *C. parapsilosis* was previously associated with a better outcome, the species of *Candida* species did not exert any influence on mortality in the present study [34].

In the majority of patients (83%) an antifungal therapy was initiated early (within 72 h from candidemia onset), and was considered appropriate in 79% of them. Interestingly, contrary to previous findings, the initiation of appropriate antifungal therapy was not associated with survival in our study [8, 10–13]. This may be attributed to the more pronounced impact of source control on outcomes. Previous studies have highlighted the role of empiric antifungal choice in patient outcomes, with echinocandins being associated with reduced mortality, especially in patients with septic shock [8, 11, 35]. However, in our study, 24% of critically ill patients received initial antifungal therapy with non-echinocandin drugs (fluconazole or liposomal amphotericin B), and this did not lead to worse outcomes compared to the group initially receiving echinocandin therapy.

While most studies have demonstrated the favorable impact of either prompt source control or early appropriate antifungal treatment on outcomes, not all have evaluated the significance of early combined management and which component is more crucial [7, 11–13, 17, 20, 21]. Previous studies have indicated that a combination of early source control and early appropriate antifungal treatment is associated with improved outcomes. In contrast, considering each intervention separately (i.e., source control or early appropriate antifungal therapy) has not consistently shown significant associations [7, 14]. Bassetti et al. demonstrated that both inadequate source control and inadequate antifungal therapy were individual predictors of worse outcomes [21]. In the present study, patients receiving both early source control and appropriate antifungal treatment exhibited comparable survival to those with only early source control. In addition, early appropriate antifungal treatment was not associated with a better outcome when compared to those without both early source control and appropriate antifungal treatment. These findings underscore the paramount importance of prompt source control in managing critically ill candidemic patients.

In the present study, SARS-CoV-2 infection was associated with increased mortality, aligning with prior reports [4, 6]. We observed a lower 30-day mortality among COVID-19 candidemic patients (36%) compared to previously reported rates (60–88%) [4, 6, 36]. An increase in incidence of candidemia among critically ill COVID-19 patients has been reported in the literature [4–6], which was more prominent in UGHP among the participating hospitals. This increased incidence may be attributed to factors such as the higher administration of immunosuppressive treatments (e.g., corticosteroids and tocilizumab) and broad-spectrum antibiotics among COVID-19 patients [36, 37].

As previously demonstrated, infection severity, as indicated by the SOFA score or the development of septic shock, was associated with mortality [10, 11, 14, 16, 17, 27]. Early source control was significantly associated with better outcome in patients with septic shock and those without. This was also shown in two previous studies with ICU candidemic patients with septic shock [7, 21]. Unlike previous research, host-related factors such as advanced age or comorbidities did not influence outcome [7, 11, 13, 15, 17].

The present study has several limitations. First, it is a retrospective study; however, it included a high number of critically ill patients from three university centers each with its distinct incidence rates, epidemiology, and clinical management practices. Second, the use of a 72-h cutoff for defining early source control and antifungal treatment may appear arbitrary. As shown in a study from LUH, approximately 30% of candidemias became positive in blood cultures after 72 h [20]. Moreover, cultures positive before 72 h faced delays in pathogen identification due to working hours, subsequently impacting source control and antifungal treatment initiation. In addition, the two Greek ICUs did not have access to rapid diagnostic tests [38]. Although beta-d-glucan was available at LUH, its usage was infrequent and, as previously demonstrated, was employed to either refrain from or discontinue empirical antifungal therapy [39]. Furthermore, no data on hydroalcoholic consumption, site of intravascular catheter insertion (jugular, subclavian, or femoral), and type of disinfection were available. Lastly, no research was conducted on the virulence or biofilm formation of different *Candida* spp.

In conclusion, this multicenter study conducted in the ICU of three university centers with varying epidemiological and clinical practices underscores the critical importance of prompt source control, particularly catheter removal in cases of catheter-related candidemia or candidemia of unknown origin. Hence, for patients diagnosed with candidemia, in addition to promptly initiating appropriate antifungal treatment, it is imperative to expeditiously undertake source control procedures, an aspect that is often overlooked in clinical practice.

## Supplementary Information

Below is the link to the electronic supplementary material.

Supplementary file1 (PDF 273 KB)

Supplementary file2 (PDF 137 KB)

## Data Availability

The data that support the findings of this study are available from the corresponding author upon reasonable request.
