# Supplementary material for: Role of source control in critically ill candidemic patients: a multicenter retrospective study
Source: Infection. 2024 Mar 12;52(5):1733–43. doi: 10.1007/s15010-024-02222-z (PMC11499412; doi:10.1007/s15010-024-02222-z)

Supplementary Figure 1. Kaplan–Meier curves illustrating the survival probabilities of patients with candidaemia in the following scenarios: (A) patients who remained under maximal care for 7 days following the onset of candidaemia, (B) patients with candidaemia of unknown origin, (C), patients with catheter-related candidaemia, and (D) patients with septic shock.

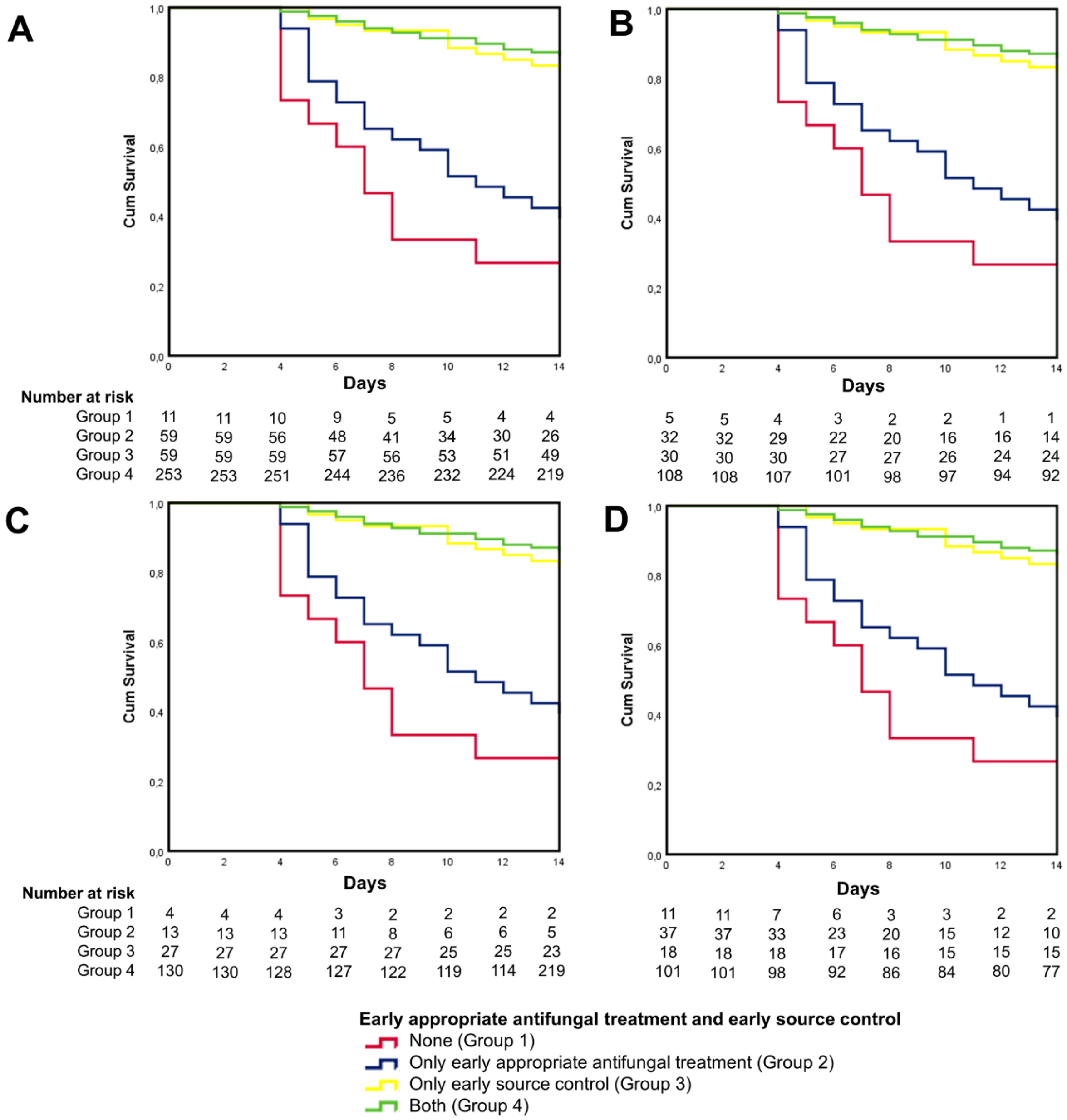

Supplement: Supplementary file 1 — Supplementary file1 (PDF 273 KB) [file 15010_2024_2222_MOESM1_ESM.pdf]
