# Supplementary material for: Role of source control in critically ill candidemic patients: a multicenter retrospective study
Source: Infection. 2024 Mar 12;52(5):1733–43. doi: 10.1007/s15010-024-02222-z (PMC11499412; doi:10.1007/s15010-024-02222-z)
Supplement: Supplementary file 2 — Supplementary file2 (PDF 137 KB) [file 15010_2024_2222_MOESM2_ESM.pdf]

**Supplementary Table 1.** Comparison of the characteristics of candidemia episodes between patients who survived and those who deceased within 30 days

| | **Survivors (n=235)** | | **Non-survivors (n=174)** | | ***P*** |
|---|---|---|---|---|---|
| Hospital | | | | | |
| LUH | 56 | 24% | 36 | 21% | |
| UGHH | 47 | 20% | 44 | 25% | |
| UGHP | 132 | 56% | 94 | 54% | 0.688[a] |
| Demographics | | | | | |
| Male sex | 170 | 72% | 105 | 60% | 0.014 |
| Age (years) | 64 | 51-73 | 69 | 61-77 | 0.012 |
| Age >60 years | 135 | 57% | 131 | 75% | <0.001 |
| Co-morbidities | | | | | |
| Congestive heart failure | 13 | 6% | 12 | 7% | 0.677 |
| Chronic obstructive pulmonary disease | 37 | 16% | 27 | 16% | 1.000 |
| Diabetes mellitus | 48 | 20% | 43 | 25% | 0.337 |
| Chronic kidney disease (moderate or severe)[b] | 14 | 6% | 21 | 12% | 0.033 |
| Malignancy (solid organ or haematologic) | 34 | 15% | 28 | 16% | 0.677 |
| Obesity | 65 | 28% | 46 | 26% | 0.823 |
| Immunosuppression[c] | 21 | 9% | 20 | 12% | 0.409 |
| Charlson Comorbidity Index | 3 | 1-5 | 4 | 3-6 | 0.023 |
| Charlson Comorbidity Index ≥4 | 99 | 43% | 102 | 58% | 0.001 |
| Microbiological data | | | | | |
| Prior episodes of candidaemia | 16 | 7% | 11 | 6% | 1.000 |
| Mixed bacterial/fungal bloodstream infection | 13 | 6% | 16 | 9% | 0.175 |
| Multiple *Candida* spp. isolated from blood cultures | 3 | 1% | 2 | 1% | 1.000 |
| *Candida* species (n=426) | | | | | |

| | | | | | |
|---|---|---|---|---|---|
| *C. albicans* | 69 | 29% | 50 | 29% | 0.913[d] |
| *Candida* non-*albicans* | 168 | 72% | 125 | 72% | |
| *C. parapsilosis* | 112 | 48% | 69 | 40% | 0.109[e] |
| *C. glabrata* | 29 | 12% | 28 | 16% | |
| *C. tropicalis* | 19 | 8% | 22 | 13% | |
| Other *Candida* spp.[f] | 9 | 4% | 7 | 4% | |
| Non susceptibility (resistance or intermediate)[g] | | | | | |
| Fluconazole | 104 | 44% | 77 | 44% | 1.000 |
| Echinocandin | 22 | 9 | 13 | 7 | 0.593 |
| Anidulafungin | 10 | 4% | 4 | 2% | 0.411 |
| Micafungin | 20 | 9% | 12 | 7% | 0.582 |
| Amphotericin B | 2 | 0.9% | 4 | 2% | 0.408 |
| Infection data | | | | | |
| Septic shock | 75 | 32% | 106 | 61% | <0.001 |
| SOFA score (points) | 8 | 5-9 | 10 | 8-12 | <0.001 |
| SOFA score ≥ 10 points | 46 | 20% | 92 | 53% | <0.001 |
| SARS-CoV-2 infection (prior month) | 47 | 20% | 53 | 31% | 0.020 |
| Breakthrough infection[h] | 123 | 52% | 91 | 52% | 1.000 |
| Infection site | | | | | |
| Unknown origin | 106 | 45% | 76 | 44% | 0.841 |
| Catheter-related | 107 | 46% | 70 | 40% | 0.313 |
| Intra-abdominal | 13 | 6% | 23 | 13% | 0.008 |
| Urinary-tract infection | 14 | 6% | 1 | 0.6% | 0.006 |
| Other[i] | 5 | 2% | 7 | 4% | 0.375 |
| Management of candidaemia | | | | | |
| Antifungal therapy initiated within 72h | 209 | 89% | 131 | 75% | <0.001 |

| | | | | | |
|---|---|---|---|---|---|
| Echinocandin (n=340) | 158 | 76% | 99 | 76% | 1.000[j] |
| Fluconazole (n=340) | 31 | 15% | 11 | 8% | |
| Liposomal amphotericin B (n=340) | 18 | 9% | 20 | 15% | |
| Appropriate antifungal therapy within 72h | 197 | 84% | 125 | 72% | 0.005 |
| Source control within 72h | 215 | 92% | 102 | 59% | <0.001 |
| Early appropriate antifungal therapy and source control | | | | | |
| None | 0 | 0% | 24 | 14% | <0.001[k] |
| Only early appropriate antifungal therapy | 20 | 9% | 48 | 28% | |
| Only early source control | 38 | 16% | 25 | 14% | |
| Both | 177 | 75% | 77 | 44% | |

Data are depicted as number and percentage for proportions or mean and SD for continuous variables

[a]Comparison UGHP against both LUH and UGHH

[b]Defined as estimated glomerular filtration rate <60 mL/min/1.73m$^2$

[c]Immunosuppression was defined as ongoing immunosuppressive treatment at infection onset, intravenous chemotherapy in the 30 days prior to infection onset, AIDS, neutropenia and asplenia.

[d]Comparison *C. albicans versus* non-*albicans*

[e]Comparison *C. parapsilosis versus* all other species

[f]*5 C. krusei, 4 C. lusitaniae, 3 C. kefyr, 2 C. dubliniensis, 2 C. guilliermondii*

[g]According to CLSI

[h]Breakthrough infection was defined as the occurrence of candidaemia in a patient having received at least three consecutive days of systemic antifungal therapy

[i]5 empyema, 4 endocarditis, 3 deep surgical site infections

[j]Echinocandin *versus* both fluconazole and liposomal amphotericin B

[k]Neither early appropriate antifungal therapy nor source control as compared *versus* all other categories

LUH: Lausanne University Hospital, SOFA: Sequential Organ Failure Assessment, UGHP: University General Hospital of Patras, UGHH: University General Hospital of Heraklion

**Supplementary Table 2.** Cox proportional hazard multivariate regression of predictors of 30-day mortality of candidaemia episodes

| | ***P*** | **HR (95% CI)** |
|---|---|---|
| Age >60 years | 0.312 | 1.24 (0.82-1.88) |
| Female sex | 0.040 | 1.39 (1.02-.90) |
| Charlson Comorbidity Index ≥4 | 0.753 | 1.06 (0.74-1.52) |
| Septic shock | 0.031 | 1.47 (1.04-2.07) |
| SOFA score ≥ 10 points | <0.001 | 2.00 (1.43-2.80) |
| SARS-CoV-2 infection (prior month) | 0.006 | 1.65 (1.16-2.35) |
| Intra-abdominal | 0.986 | 1.04 (0.62-1.63) |
| Early appropriate antifungal therapy and source control | | |
| None | reference | reference |
| Only early appropriate antifungal therapy | 0.226 | 1.33 (0.84-2.09) |
| Only early source control | <0.001 | 0.27 (0.18-0.39) |
| Both | <0.001 | 0.12 (0.07-0.21) |

HR: hazard ratio; SOFA: Sequential Organ Failure Assessment
